# Breast MRI quality assurance in practice

**DOI:** 10.1186/bcr3313

**Published:** 2012-11-09

**Authors:** S Bacon, B Dall, N Sharma, D Manuel, D Wilson

**Affiliations:** 1 St James's Institute of Oncology, Leeds, UK

## Introduction

Breast MRI has been incorporated into the NHSBSP programme and therefore is subject to quality assurance (QA) to NHSBSP standards. The breast screening technical guidelines recommend weekly testing of signal-to-noise ratio (SNR) and suppression effectiveness. We tested a method of implementing these recommendations on a Siemens 1.5T Avanto scanner using phantoms and software supplied as standard.

## Methods

Phantoms were placed in the right and left apertures of the Siemens four-element breast matrix coil and manufacturer QA performed. Suppression effectiveness was measured by acquiring a 3D spoiled gradient echo (FLASH) sequence with and without water suppression. Regions of interest (ROI) were drawn in both phantoms and percentage signal reduction due to water suppression calculated for three slices. SNR was measured using the standard fat-suppressed 3D FLASH sequence acquired three times after waiting 10 minutes to minimise fluid motion artefacts. Subtractions were performed and ROI drawn in both phantoms. SNR was calculated as √2×(mean signal intensity)/(standard deviation of the subtraction image) for three slices.

## Results

The breast coil passed the manufacturer QA tests. Mean suppression effectiveness was 97.0% (95.6 to 98.0%) for the right phantom and 96.9% (95.4 to 97.7%) for the left. Mean SNR was 98.8 (41.2 to 110.9) for the right phantom and 96.2 (53.8 to 115.4) for the left. The low values of SNR were likely to be due to artefacts from fluid motion on the subtraction images used for noise calculations.

## Conclusion

The methods proposed allowed an independent measure of suppression effectiveness. More work is needed to ensure a reliable measurement of SNR.

